# Editorial: Glycolipid metabolism disorders in cerebrovascular and cardiovascular diseases: advanced insights into pathology, pathophysiology and treatment

**DOI:** 10.3389/fmed.2025.1586403

**Published:** 2025-03-17

**Authors:** Zhengyang Bao, Rong Jin, Haijian Sun

**Affiliations:** ^1^Affiliated Wuxi Maternity and Child Health Care Hospital of Jiangnan University, Wuxi, China; ^2^Wuxi School of Medicine, Jiangnan University, Wuxi, China; ^3^Department of Neurosurgery, Pennsylvania State University College of Medicine, Hershey, PA, United States

**Keywords:** cerebrovascular diseases, cardiovascular diseases, glycolipid metabolism, pathological mechanism, treatment

Glycolipid metabolism disorders, characterized by dysregulated lipid and glucose homeostasis, are increasingly recognized as critical contributors to cerebrovascular and cardiovascular pathologies, including atherosclerosis, ischemic stroke, myocardial infarction, and vascular calcification (1–3). The intricate interplay between metabolic dysregulation and cardiovascular dysfunction underscores the urgency of elucidating these mechanisms to advance diagnostic and therapeutic innovations.

In the current article collection on the *Frontiers in Medicine* Research Topic “*Glycolipid metabolism disorders in cerebrovascular and cardiovascular diseases: advanced insights into pathology, pathophysiology, and treatment*,” we highlight several articles and reviews to provide novel insights into the relationship between glycolipid metabolism disorders and cerebrovascular and cardiovascular Diseases.

Jiang et al. investigated the relationship between the triglyceride glucose-body mass index (TyG-BMI) and coronary artery calcification (CAC) in maintenance hemodialysis (MHD) patients. Their findings identified TyG-BMI as a significant independent predictor of CAC progression, establishing a direct association between glycolipid metabolic dysfunction and vascular calcification. By integrating insulin resistance markers with lipid-glucose homeostasis indicators, this study underscores the critical role of metabolic dysregulation in cardiovascular complications. These insights lay the foundation for refining risk stratification protocols and developing targeted therapeutic strategies for patients with glycolipid metabolism disorders.

In another study, Su X. et al. conducted a cross-sectional study titled “*Association between METS-IR and heart failure: a cross-sectional study*,” using data from the National Health and Nutrition Examination Survey (NHANES). The research revealed a J-shaped relationship between the Metabolic Score for Insulin Resistance (METS-IR) and heart failure (HF) risk. Mechanistic analyses highlighted insulin resistance is a key driver of HF pathogenesis, with pathways involving lipotoxicity-induced cardiomyocyte apoptosis, chronic low-grade inflammation, and impaired myocardial energy metabolism. These findings underscored METS-IR is a promising biomarker for identifying high-risk populations with metabolic dysregulation and facilitating early intervention in glucose-lipid homeostasis management (Su X. et al.).

Zhu et al.'s study, based on NHANES data, found a significant association between the Cardiometabolic Index (CMI) and heart failure, highlighting the critical role of glucose and lipid metabolism disorders in the development of heart failure. As an integrated indicator of metabolic health, CMI combines blood glucose, lipids, waist circumference and other parameters to comprehensively reflect individual metabolic abnormalities. Their results showed that higher CMI levels were associated with an increased risk of heart failure, suggesting that glucose and lipid metabolism disorders are not only closely related to cardiovascular dysfunction but may also accelerate the progression of heart failure through various mechanisms. Moreover, compared to individual metabolic markers, CMI preferably captures the long-term impact of metabolic abnormalities on cardiac health by integrating multiple features of metabolic syndrome. Therefore, CMI could serve as an effective clinical tool to identify high-risk individuals with metabolic disorders, offering valuable guidance for the prevention and management of heart failure (Zhu et al.).

Lidón-Muñoz et al. highlighted the link between therapeutic adherence and cardiovascular disease, emphasizing the critical role of glucose and lipid metabolism disorders in secondary prevention. Poor adherence to treatments like statins and antihypertensives worsens metabolic dysregulation, increasing cardiovascular risks. An intervention based on the Chronic Care Model aims to improve adherence, empowering patients to manage metabolic disorders and reduce recurrent cardiovascular events (Lidón-Muñoz et al.).

Su J. et al. discussed the critical role of the cGAS-STING signaling pathway in linking inflammation with metabolic disorders, particularly through its impact on glucose and lipid metabolism. Activation of this pathway is associated with mitochondrial stress, leading to the release of mitochondrial DNA (mtDNA), which in turn triggers inflammatory responses that exacerbate insulin resistance, obesity, and non-alcoholic fatty liver disease (NAFLD). In hepatocytes, cGAS-STING activation promotes lipid accumulation and fibrosis, while it reduces thermogenesis and increases fat storage in adipose tissue, creating a feedback loop of metabolic dysregulation. These findings suggest that cGAS-STING dysregulation not only drives chronic inflammation but also directly disrupts glucose and lipid homeostasis, making it a potential therapeutic target for metabolic diseases (Su J. et al.).

This Research Topic also underscores the translational potential of advanced diagnostics and therapeutic strategies. Emerging tools such as lipidomic profiling and novel biomarkers are paving the way for early detection and personalized interventions. Therapeutic approaches targeting insulin resistance and inflammation offer promising avenues for mitigating cardiovascular complications.

We extend our deepest gratitude to the authors, reviewers, and editorial team for their valuable contributions. We hope that this Research Topic inspires further research and innovation in unraveling the complexities of glycolipid metabolism disorders and their impact on cerebrovascular and cardiovascular ailments.
